# Amalric sign and central retinal artery with lateral posterior ciliary artery occlusion

**DOI:** 10.4103/0301-4738.55060

**Published:** 2009

**Authors:** Natesh Sribhargava, K Harsha, Savitha Prasad, Bhujang K Shetty

**Affiliations:** Department of Ophthalmology, Narayana Nethralaya

Dear Editor,

Amalric was the first to describe an unusual triangular pigmented disturbance in the fundi of patients with ischemic ocular disorders.[1] The same sign is described in vasculitides like polyarteritis nodosa,[2] giant cell arteritis and Wegener's granulomatosis. We present our experience of managing a patient with “Amalric sign”.

A 37-year-old gentleman, a chronic smoker, presented with sudden onset of blurred vision in left eye of three days duration. Best corrected vision in right eye was 20/40 and left eye was counting fingers at half meter.

**Figure 1a,1b:**
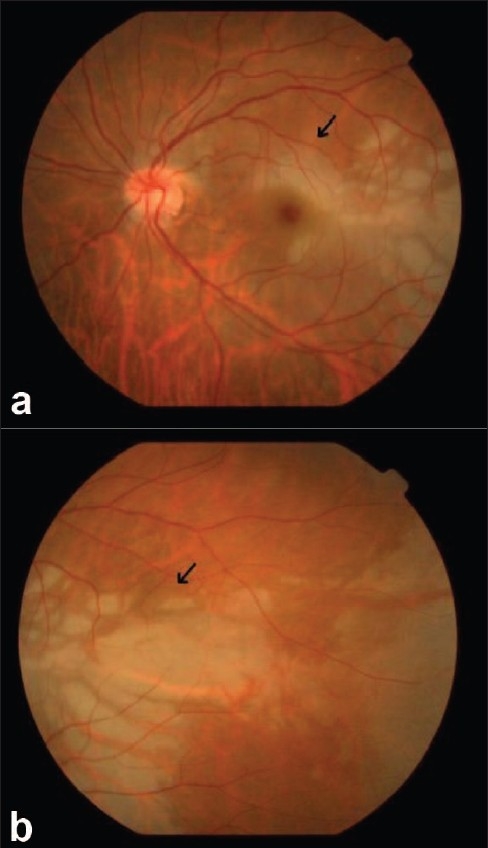
Pigmentary changes at midperiphery temporal to the macula

**Figure 2:**
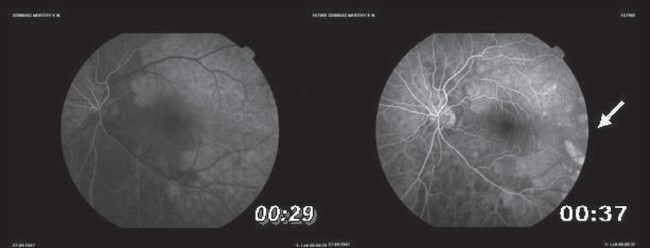
Fundus fluorescein angiography depicting delayed arteriovenous transit time and the hyperfluorescent areas at the midperiphery temporal to the macula

**Figure 3a and 3b:**
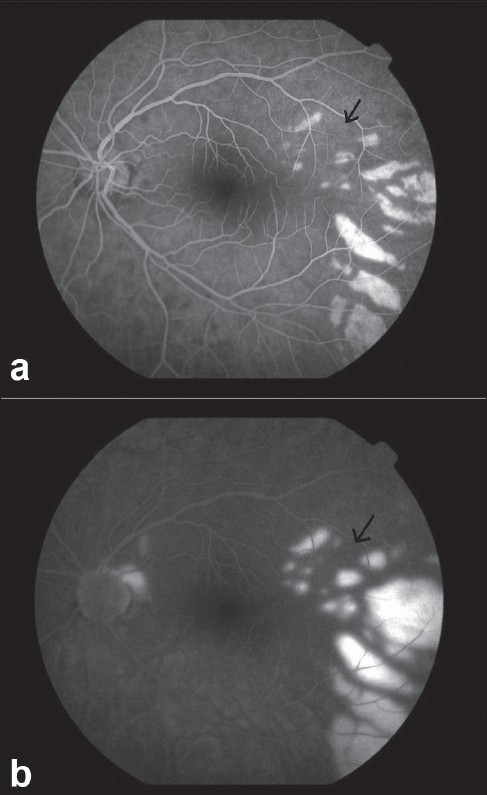
Late phases of angiography showing the typical triangular hyperfluorescent area

**Figure 4a and 4b:**
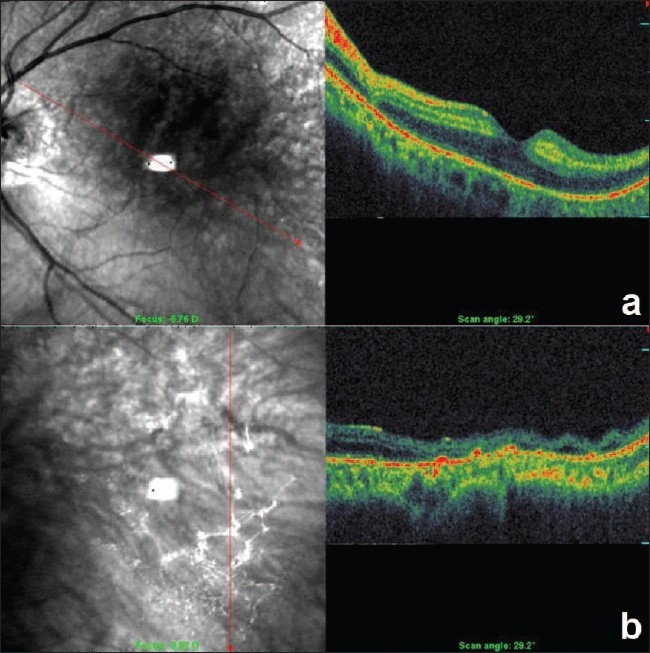
Optical coherence tomography (OCT) picture through the fovea (4a), OCT through the hypopigmented lesion at the midperipheral area showing thinning of the retina (4b)

He had a relative afferent pupillary defect (RAPD) in left eye, and retinal edema with an indistinct cherry red spot. There were triangular hypopigmented subretinal lesions extending temporal to the macula [Fig. 1a,1b]. Fundus fluorescein angiography showed delayed arm to retina time, widened watershed zone, choroidal non-perfusion areas, delayed arteriovenous transit time and triangular hyperfluorescent areas corresponding to the hypopigmented patches which densely fluoresced in late phase [Fig. 2,3a, 3b]. Optical coherence tomography showed increased reflectivity and thickness of the inner retina and a corresponding decrease of reflectivity in the outer retinal layers. The retina was thin over the hypopigmented lesions [Fig. 4a, 4b]. Investigations done were normal except for raised triglycerides 700 mg/dL (ref: 40-140), decreased high-density lipoprotein 12 mg/dL (ref: 30-60) levels and elevated serum homocysteine levels 27.47 mcmol/L (ref: 5.90-16.0). Echocardiography was normal. Carotid Doppler showed complete occlusion of left distal internal carotid artery (ICA).

This case of central retinal artery occlusion (CRAO) with lateral posterior ciliary artery (LPCA) and distal ICA occlusion demonstrates the rare finding of triangular sign of Amalric. Amalric postulated that this disturbance was caused by choroidal infarction.[1] Hayreh produced similar lesions in rhesus monkeys by cutting the medial and LPCA. He noted that these lesions occurred at 18–24 h of PCA occlusion, were elongated, subretinal and became depigmented after two to three weeks.[3] They were located in the periphery, with bases toward the equator and apices toward the posterior pole.[4]

This is the first photographed case of the triangular sign of Amalric in CRAO and LPCA occlusion due to ICA occlusion in a smoker. The delayed arteriovenous transit time, RAPD suggest CRAO apart from the cherry red spot. The patient did not have any optic disc features of anterior ischemic optic neuropathy. The lacunae were the lack of indocyanin green (ICG) and electrophysiological tests. However, patient did not consent for the same and was lost to follow-up.

Amalric sign is a rare clinical finding that indicates choroidal ischemia and may be associated with CRAO.
